# Outbreak of human leptospirosis linked to contaminated water bodies in Northern Israel, June to August 2018

**DOI:** 10.2807/1560-7917.ES.2018.23.38.1800486

**Published:** 2018-09-20

**Authors:** Yuval Dadon, Eric J. Haas, Ehud Kaliner, Emilia Anis, Shepherd Roee Singer, Yafit Atiya-Nasagi, Michal Cohen-Dar, Eva Avramovich, Roni King, Oded Sued, Tamir Goshen, Sharon Amit, Ian Miskin, Efrat Gino, Ruth Yishai, Rivka Sheffer, Itamar Grotto, Jacob Moran-Gilad

**Affiliations:** 1 Ministry of Health Directorate, Jerusalem, Israel; 2 The Division of Epidemiology, Public Health Services, Ministry of Health, Jerusalem, Israel; 3 These authors contributed equally to the study; 4 School of Public Health, Faculty of Health Sciences, Ben-Gurion University of the Negev, Beer-Sheva, Israel; 5 Public Health Services, Ministry of Health, Jerusalem, Israel; 6 Braun School of Public Health and Community Medicine, Hebrew University and Hadassah, Jerusalem, Israel; 7 Israel Institute for Biological Research, Ness Ziona, Israel; 8 Northern District, Ministry of Health, Israel; 9 Public Health Branch, IDF Medical Corps, Israel; 10 Nature and Parks Authority, Jerusalem, Israel; 11 Mekorot Central Laboratory, Eshkol Site, National Water Company, Israel; 12 Israeli Veterinary Services, Ministry of Agriculture and Rural Development, Beit Dagan, Israel; 13 Hadassah Medical Center, Jerusalem, Israel; 14 Jerusalem District, Clalit Health Services, Jerusalem, Israel; 15 Public Health Laboratory, Haifa District, Ministry of Health, Haifa, Israel

**Keywords:** leptospirosis, waterborne, travel, climate change, contamination, outbreak investigation

## Abstract

We report preliminary findings of a large outbreak of human leptospirosis with 36 confirmed/probable and 583 suspected cases from June–August 2018, linked to contaminated water bodies in Northern Israel. There was a travel-associated case in Germany; additional cases are being investigated in other countries. The presumed chain of transmission, implicating wild boar and cattle, raises multiple challenges for risk assessment, risk management and risk communication currently being addressed by a public health response team.

Leptospirosis is a common zoonotic disease worldwide, especially in tropical areas, which is associated with human exposure to pathogenic *Leptospira*. Here we describe an ongoing outbreak investigation and the public health response to human leptospirosis cases linked to exposure to contaminated recreational water bodies in Northern Israel.

## Outbreak detection

In 9 August 2018, 15 cases, including a group of soldiers travelling together, with an acute febrile syndrome consistent with acute leptospirosis were notified from various hospitals across Israel, instigating an outbreak investigation. A potential common exposure was quickly recognised as being linked to recreational activities (including water rafting or kayaking) in several popular water bodies in Northern Israel, during the 3 weeks before onset of illness. Of note is that the implicated water bodies are heavily attended each year by the public in July and August. During recent years and especially in 2018, multi-year drought conditions resulted in seasonally low water levels in the region. Public water bodies in Israel are routinely monitored and inspected for faecal contamination according to national regulations for recreational water quality and in the past have been required to close to the public due to abnormal faecal contamination levels during the summertime.

## Immediate public health response

The public health response, led by the Public Health Services of the Israeli Ministry of Health (MOH), included the formation of a multi-disciplinary outbreak control team. This team included a range of experts and stakeholders such as infectious disease specialists, epidemiologists, microbiologists, the Veterinary Services at the Ministry of Agriculture, the Nature and Parks Authority and the National Water Authority and Water company (Mekorot) and communication specialists. An outbreak case definition formulated collaboratively by the outbreak control team can be seen in the Box.

Box **Case definition for leptospirosis outbreak, formulated by the outbreak control team, Israel, 14 August, 2018**

**Suspected cases^a^**: individuals with any acute febrile illness accompanied by relevant symptoms or signs (modified from United States Centers for Disease Control and Prevention [1]), occurring within 2 days to 3 weeks of recent exposure to the relevant water bodies^b^.

**Probable cases**: suspected cases with a borderline-positive serology to leptospirosis (1:50 to 1:150).

**Confirmed cases**: suspected cases with laboratory confirmation of leptospirosis using molecular techniques (positive real-time polymerase chain reaction (PCR) for pathogenic leptospires applied on blood or urine samples) and/or serology (positive microagglutination test (MAT), using a panel of 24 antigens, defined as a single titre of 1:200 or more or a fourfold rise of titre over 10 or more days).

^a^At the time of writing, convalescent-phase sera have not yet been received and tested for the vast majority of cases and since initial negative PCR and/or MAT cannot rule out leptospirosis, patients meeting the case definition but with negative serology were classified as ‘suspected’ and not excluded.
^b^The case definition initially included four body sites (Zavitan, Yehudiya, Meshushim and Zaki). Three additional sites were added a few days later following the recognition of additional exposure sites.

Active retrospective and prospective surveillance of leptospirosis cases was initiated. The MOH notified the public through various media channels about the outbreak and recommended that individuals meeting the case definition seek medical advice in community clinics or hospitals. The MOH issued detailed guidance for healthcare professionals concerning the diagnosis, treatment alternatives and reporting of suspected leptospirosis in the community and hospital settings. Empiric age-based antimicrobial therapy was recommended for suspected cases at the discretion of attending physicians. Antimicrobial prophylaxis and personal protective equipment were recommended for potential occupational exposures (e.g. water inspectors in the region). The seven water bodies linked to suspected leptospirosis cases were closed to the public until further notice and the closure was publicised through a variety of media channels.

## Public health microbiology response

Based on continuous risk assessment, the MOH increased the frequency of recreational water quality sampling to two to three times per week at the implicated sites (Public Health Laboratory, Haifa), as well as in a range of other water bodies in Northern Israel where human illness had not been reported. Under the assumption (and in line with the epidemiological hypothesis) that faecal coliforms could serve as a surrogate marker for animal-derived contamination of water, the microbial thresholds for site closure were lowered from 1,000 to 400 colony-forming units (cfu)/100mL of faecal coliforms. The National Reference Laboratory for Leptospirosis (Israel Institute for Biological Research (IIBR), Ness Ziona), mounted a surge response in order to process a large number of clinical samples by means of a duplex real-time polymerase chain reaction (PCR) for pathogenic leptospires (targeting the 16S rRNA and the *LipL32* genes) and microagglutination test (MAT). A real-time PCR testing of water for the implicated water bodies has also been performed on a ‘research use only’ basis. In addition, a series of quantitative molecular tests using the microbial source tracking (MST) strategy for detection of faecal contamination source was deployed (Mekorot Central Laboratory) [2].

## Outbreak description

**Figure 1:**
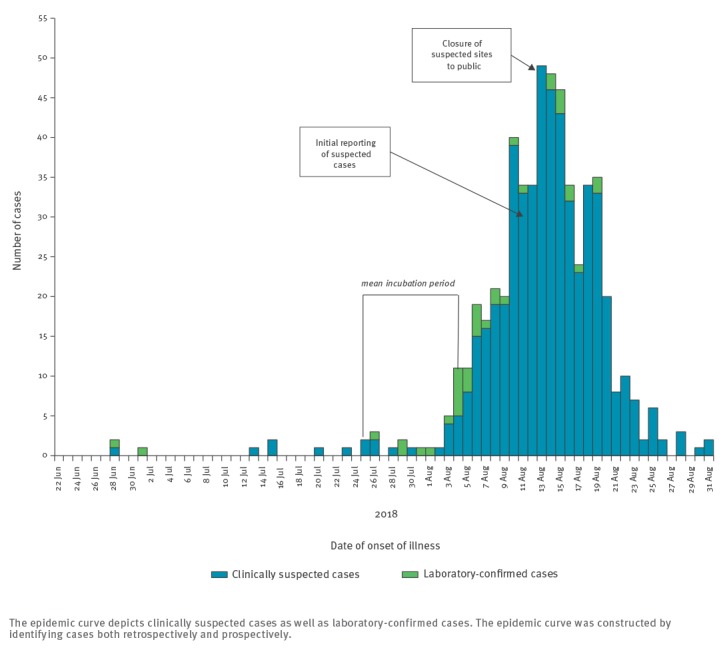
Epidemic curve for human leptospirosis in Northern Israel, June–August 2018 (n = 619)

As of 31 August 2018, 619 suspected cases of leptospirosis had been notified to the MOH, of which 33 were laboratory-confirmed and three were probable. The remaining cases had initial negative MAT and/or PCR (leptospirosis not ruled out). The epidemic curve of the outbreak is shown in Figure 1. 

The epidemic curve depicts clinically suspected cases as well as laboratory-confirmed cases. The epidemic curve was constructed by identifying cases both retrospectively and prospectively.

Retrospective case finding identified a confirmed case of infection afflicting a water inspector dating back to June 2018. The characteristics of laboratory-confirmed cases are summarised in Table 1. Of the 33 laboratory-confirmed and three probable cases, 31 were male and the median age was 20 years (mean 25, range 7-64). The incubation period ranged from 2–21 days (mean 9.9 days, median 10 days). Influenza-like symptoms were common while rash and conjunctivitis were rare. The majority (n = 31) of confirmed cases were hospitalised, 12 had hepatic or renal involvement and one patient developed pneumonia. Three were reported to have been admitted to an intensive-care unit; no deaths were reported. There was one travel-associated case reported in a German tourist who developed severe leptospirosis [3], it is thought that this case was exposed in early June.

**Table:** Characteristics of laboratory-confirmed and probable cases of leptospirosis, Israel, June–August 2018 (n = 36)

Date of onset of illness in 2018	Incubation period (days)	Relevant exposure	Clinical symptoms	Illness severity	Laboratory results	Case-classification
28 June	13	Meshushim pool	Fever, chills, weakness, myalgia	Hospitalised	MAT ≥ 1:200	Confirmed
1 July	9	Jordan Park / Arik Bridge	Fever, weakness, headache, myalgia, vomiting	Hospitalised, renal failure	MAT ≥ 1:200	Confirmed
26 July	2	Meshushim pool	Fever, chills, weakness, headache, myalgia	Hospitalised, renal failure, elevated liver enzymes	MAT = 1:100	Probable
29 July	2	Zavitan stream	Fever, weakness, headache, myalgia, vomiting	Hospitalised, elevated creatinine and liver enzymes	MAT ≥ 1:200	Confirmed
31 July	2	Jordan Park / Arik Bridge	Fever, weakness, headache, myalgia	Hospitalised	Positive PCR	Confirmed
1 August	7	Zavitan stream	Fever, chills, weakness, headache, myalgia	Hospitalised, renal failure, elevated liver enzymes	MAT ≥ 1:200	Confirmed
3 August	10	Yehudiya forest reserve	Fever, chills, weakness, headache	Hospitalised	MAT ≥ 1:200	Confirmed
4 August	12	Yehudiya forest reserve	Fever, weakness, headache, vomiting	Hospitalised	MAT ≥ 1:200	Confirmed
4 August	8	Meshushim pool	Fever, chills, weakness, headache, conjunctivitis, myalgia, cough, vomiting, diarrhoea	Hospitalised, renal failure, elevated liver enzymes	MAT ≥ 1:200	Confirmed
4 August	7	Yehudiya forest reserve	Fever, headache, myalgia, nausea,	Hospitalised, pneumonia	MAT ≥ 1:200	Confirmed
4 August	6	Meshushim pool	Fever, chills, weakness, headache, myalgia	Hospitalised	MAT ≥ 1:200	Confirmed
4 August	11	Meshushim Pool	Fever, headache	Emergency department, immunocompromised at baseline	MAT > 1:200	Confirmed
4 August	10	Meshushim Pool	Fever, chills, weakness, headache, myalgia,	Ambulatory	MAT > 1:200	Confirmed
5 August	12	Yehudiya forest reserve	Fever, chills, weakness, headache, myalgia	Hospitalised, renal failure and elevated liver enzymes	Positive PCR, MAT ≥ 1:200	Confirmed
5 August	9	Meshushim pool	Fever, headache, myalgia vomiting, rash	Hospitalised	MAT 1:100	Probable
5 August	9	Zaki stream	Fever, weakness, myalgia, vomiting	Hospitalised	Positive PCR, MAT ≥ 1:200	Confirmed
5 August	5	Meshushim pool	Pending investigation	Pending investigation	MAT ≥ 1:200 (seroconversion)	Confirmed
6 August	19	Zaki stream	Fever, chills, weakness, headache, myalgia	Hospitalised	MAT ≥ 1:200	Confirmed
6 August	14	Yehudiya forest reserve	Fever, chills, weakness, headache, myalgia	Hospitalised	MAT ≥ 1:200	Confirmed
6 August	2	Yehudiya forest reserve	Fever, weakness, myalgia, headache, vomiting, diarrhoea, leukopenia, thrombocytopenia	Hospitalised, intensive care	Positive PCR	Confirmed
7 August	10	Meshushim pool	Fever, headache, myalgia, jaundice	Hospitalised	MAT ≥ 1:200 (seroconversion)	Confirmed
8 August	4	The Majrasa	Fever, chills, weakness, headache, myalgia	Hospitalised, intensive care	Positive PCR	Confirmed
8 August	15	Zaki stream	Fever, weakness, headache, myalgia	Hospitalised, renal failure, elevated liver enzymes	MAT ≥ 1:200	Confirmed
9 August	9	Meshushim pool	Fever, weakness, headache, myalgia, arthralgia	Emergency department	Positive PCR	Confirmed
10 August	12	Meshushim pool	Fever, weakness, headache, myalgia, vomiting, diarrhoea	Hospitalised	Positive PCR	Confirmed
11 August	12	The Majrasa	Fever, chills, weakness, headache, myalgia, vomiting	Hospitalised, intensive care	MAT = 1:50	Probable
14 August	19	Yehudiya forest reserve	Pending investigation	Pending investigation	MAT > 1:200	Confirmed
14 August	11	Jordan Park / Arik Bridge	Fever, chills, weakness, headache, myalgia	Hospitalised	Positive PCR	Confirmed
15 August	21	Yehudiya forest reserve	Fever, chills, weakness, myalgia, conjunctivitis	Hospitalised, elevated liver enzymes	Positive PCR	Confirmed
15 August	10	Jordan Park / Arik Bridge	Fever, chills, weakness, headache, jaundice	Hospitalised, elevated liver enzymes	Positive PCR	Confirmed
15 August	13	Zavitan Stream	Fever, chills, weakness, headache, myalgia, vomiting	Hospitalised	Positive PCR	Confirmed
16 August	10	Gilabon Stream	Fever, weakness, headache, myalgia, vomiting	Hospitalised	Positive PCR	Confirmed
16 August	6	Meshushim Pool	Fever, chills, myalgia, vomiting, conjunctivitis	Hospitalised	Positive PCR	Confirmed
17 August	8	Gilabon Stream	Fever, weakness, headache, vomiting	Hospitalised	Positive PCR	Confirmed
19 August	9	Jordan Park / Arik Bridge	Fever, chills, weakness, headache, myalgia, diarrhoea, conjunctivitis	Hospitalised, renal failure	Positive PCR	Confirmed
19 August	17	Gilabon Stream	Fever, chills, weakness, myalgia	Hospitalised, renal failure	Positive PCR	Confirmed

MAT: microagglutination test; PCR: Polymerase chain reaction.

**Figure 2:**
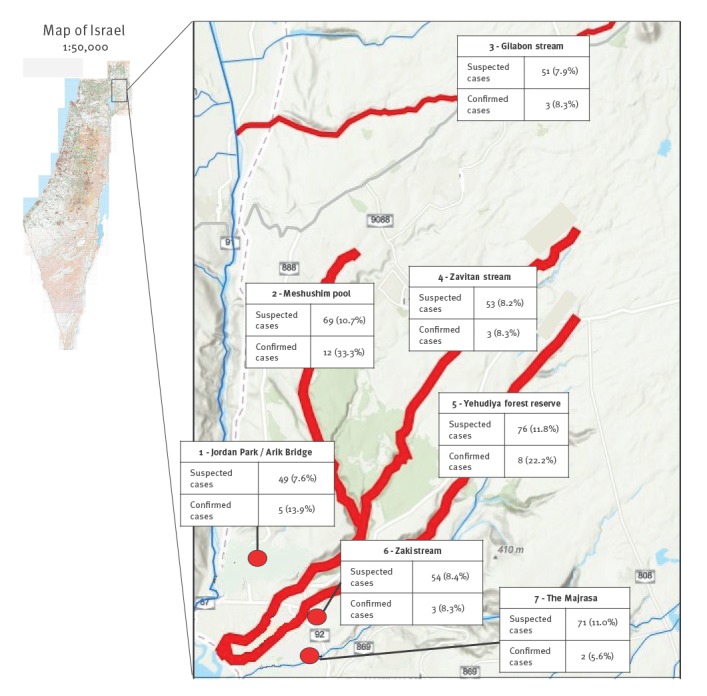
Spatial distribution of leptospirosis cases in Northern Israel, June–August 2018 (n = 540)

The geographic distribution of cases is shown in Figure 2. Exposure data were available for 540 cases (87.2%); all 36 confirmed and probable cases had exposures to one or more of seven recreational water bodies in the weeks before onset of illness; for the remaining 504 suspected cases, 645 potential exposures were noted, of which 423 (65.5%) involved the implicated water bodies. 

Spatial representation of seven water sources implicated in acquisition of leptospirosis in Northern Israel. All 36 confirmed/probable cases were linked to at least one of the seven sites (5.6-33.3%). Among the remaining 504 suspected cases, there were 423 potential exposures to these seven sites (7.6-11.8%). The figure zooms in on the region north-east to the Sea of Galilee. Red lines represent contaminated streams.

## Environmental investigation

**Figure 3:**
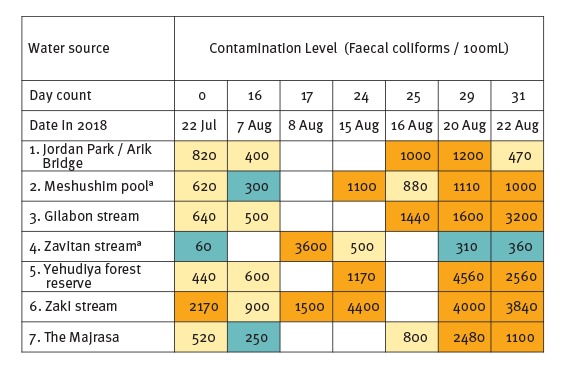
Faecal contamination of water bodies in Northern Israel, June–August 2018

^a^Water bodies tested positive for pathogenic *Leptospira* by real-time PCR.

Laboratory surveillance for faecal contamination (Figure 3) revealed abnormal faecal coliform counts on repeated measurements from all water bodies, supporting the decision for site closure to the public. Subsequent MST of the initial four water sources identified heavy faecal contamination attributed to wild boar and cattle, with only a trace of human faeces. Positive serological tests by MAT were reactive against a range of serovars as commonly seen in early leptospirosis. The Pomona and Burgas antigens (Serovars Pomona and Balcanica, respectively) were particularly reactive (seven and ten cases, respectively) with titres ranging from 1:200 to 1:1,600. The Pomona serovar, in particular, is naturally adapted to wild boar and there have been recent reports from the Israeli veterinary authorities on the emergence of *L*. Pomona infection in wild boar and cattle in Northern Israel [4]. Faecal contamination of water bodies from wild boar and cattle origin, in combination with clinical infection with the Pomona serovar, support human infection acquired through exposure to animal excretions during recreational activity under favourable conditions for transmission, i.e. drought and relative water stagnation.

Serial faecal coliform counts (colony forming units (cfu)/100mL) used as a surrogate marker for water contamination with animal excretions in the seven water sources implicated in acquisition of leptospirosis in Northern Israel. Green, yellow and orange denote counts of < 400, 400–1,000 or > 1,000, respectively, per regulatory thresholds used for risk assessment and risk management.

## Discussion

The severity of leptospirosis varies greatly, ranging from asymptomatic infection to mild influenza-like illness to a severe life-threatening infection (Weil’s disease) [5]. The severity of infection depends on the type of exposure, the human host and the infecting serovar. Human infection commonly involves direct or indirect exposure to infected urine originating from animal reservoirs, this can occur both recreationally or occupationally [6]. While many cases are sporadic, outbreaks of human leptospirosis have been occasionally reported.

Human leptospirosis is uncommon in Israel; data from the MOH surveillance of notifiable diseases suggest less than 10 confirmed cases have occurred per annum over the last decades [7]. An outbreak of seven cases of leptospirosis acquired during combat training of soldiers was reported in 2002 and was caused by a low pathogenicity serovar (*L*. Hardjo) commonly found in cattle in Israel [8]. The current outbreak is much larger and subject to ongoing investigation and case surveillance. To date it has involved considerable communication challenges, with respect to the risk of contracting leptospirosis in certain locations, while providing assurance for the safety of continued recreational activities in the region as a whole. Difficulty in obtaining accurate travel history for cases in the weeks following possible exposure was particularly challenging. To overcome challenges during this outbreak, communication strategies involved not only national web-based, electronic and printed media, but also externalisation of visual aids based on geographic information systems (GIS). Since the North of Israel is a popular site for domestic and international tourism, especially during the summer, cases may show up elsewhere in returning travellers. Individuals meeting the case definition may therefore be considered as suspected cases.

In addition, the relevant government agencies have used water originating from other regional water sources to help increase the water flow to the implicated water bodies; thus, reducing stagnation and helping to dilute and wash away faecal contamination. To date, four sites have been targeted for increased water flow, while monitoring of effectiveness continues using serial faecal coliform as well as MST testing. In the absence of validated regulatory tests for environmental contamination with pathogenic *Leptospira*, the criteria for re-opening of water bodies for the public visit are complex. Currently, they rely on a combination of epidemiological data and direct and indirect microbiological tests as part of an ongoing risk assessment.

Long-term solutions are currently being considered to prevent further water contamination. These include improved control of animal leptospirosis in ruminants, through employing repeated testing, antimicrobial treatment and vaccination with polyvalent vaccines containing the implicated serovar. In addition, solutions for prevention of further contamination by providing designated water sources (basins) and limiting natural water access of free-ranging animals are currently being considered and involve a cross-sectoral effort of multiple government offices.
